# Malnutrition is associated with increased disease risk in older people in the Makkah region of Saudi Arabia: A cross-sectional study

**DOI:** 10.3389/fpubh.2023.1149739

**Published:** 2023-04-03

**Authors:** Maha A. Althaiban, Najlaa M. Aljefree, Noha M. Almoraie, Israa M. Shatwan

**Affiliations:** Food and Nutrition Department, Faculty of Human Sciences and Design, King Abdulaziz University, Jeddah, Saudi Arabia

**Keywords:** malnutrition, aging, older people, overweight, nutritional status, depression, eating disorders, Saudi Arabia

## Abstract

**Introduction:**

There is little research on the nutritional status of older people in Saudi Arabia. This study investigated the factors associated with the nutritional status of older people in the Makkah region, Saudi Arabia. We hypothesized that older people who are at risk of malnutrition are at higher risk of different diseases.

**Materials and methods:**

This cross-sectional study surveyed 271 people aged ≥60 years from October 2021 to January 2022. We collected data on demographics, body mass index, the Geriatric Depression Scale-Short Form, Geriatric Oral Health Assessment Index, Mini Nutritional Assessment, Eating Attitudes Test, and Household Dietary Diversity score.

**Results:**

Among the 271 participants, 13.3% were malnourished and 53.9% were at risk of malnutrition. The oral health (*P* < 0.001), depression (*P* < 0.001), and eating disorder (*P* < 0.002) scores were significantly associated with malnutrition. Congestive heart failure, asthma, peripheral vascular disease, Alzheimer's disease, and hypertension were more prevalent among malnourished participants—this supports our original hypothesis. The HDD score showed no significant differences between men and women.

**Conclusion:**

Malnutrition was associated with overweight or obesity, poor oral health, and depression. Older people in the Makkah region, Saudi Arabia, had a high risk of malnutrition.

## 1. Introduction

Health problems related to malnutrition have increased worldwide in the older population. Malnutrition refers to insufficient (undernutrition) or excess (overnutrition) nutrient intake that contributes to health conditions such as obesity, diabetes, and coronary heart disease (1, 2). Studies have reported that malnutrition is significantly associated with hospitalization in older people, and ~90% of those in hospitals are malnourished or at risk of malnutrition (3). In Australia, 42.3% of older adults admitted to hospitals are malnourished (4). Many psychological and biological changes, such as loss of taste and smell sensitivity, chewing difficulties due to missing teeth, and impaired activities of daily living (5), which affect the intake of certain foods and nutrients are associated with old age and increase the risk of nutrition-related health problems.

Globally, the older population is rapidly expanding (6, 7), with individuals aged ≥65 years accounting for 10% of the global population (1). According to the World Health Organization (WHO), the older population will reach 1.2 billion by 2025, with most residing in low-income countries (7). Furthermore, it has been suggested that by 2025, the percentage of older people worldwide will increase by 300% above that in 2000 (8). With improved medical care and living conditions, Saudi Arabia's demographics are slowly but steadily changing, with life expectancy increasing in recent decades (8). *Here, an estimated 4.3% of the population were aged 55–64 years in 2013, while it is predicted that 18.4% will be aged* ≥*65 years in 2050* (9). Saudi Arabia has witnessed remarkable development in socioeconomic status in the past 40 years, combined with significant lifestyle changes, such as the adoption of Western dietary patterns and reduction in physical activity levels, which has led to a high prevalence of chronic diseases and nutrition-related health problems among different age groups, including older people (10).

Malnutrition increases the risk of morbidity and mortality in the older population (11). Previous research has shown that malnutrition is associated with several factors, including socioeconomic factors (education, sex, and marital status) (12, 13), eating habits (14), and psychological (depression and dementia) (15) and medical factors (osteoporosis, dysphagia, and oral health problems) among older people (16–18). Hence, early detection and treatment of malnutrition in older people is critical for reducing morbidity and improving quality of life. Malnutrition has been reported to be prevalent in 25.6% of older adults in Bangladesh (19) and 17.9% in India (6). However, a previous study showed that only 5.8% of older adults in developed countries, including Switzerland, Sweden, and Japan, are malnourished (20). There is a paucity of research on the nutritional status and factors associated with malnutrition among older people in Saudi Arabia. Therefore, this study aimed to address this gap and investigate the factors associated with the nutritional status of the older population in the Makkah region of Saudi Arabia. We hypothesized that older people who are at risk of malnutrition are at higher risk of different diseases.

## 2. Materials and methods

### 2.1. Study design and participants

This cross-sectional study surveyed 271 men and women aged ≥60 years in the two largest cities in the Makkah region of Saudi Arabia, Makkah and Jeddah, from October 2021 to January 2022. As defined by the United Nations, individuals aged ≥60 years were considered as the older population (21). According to the General Authority for Statistics, a total of 321 769 people (both Saudis and non-Saudis) aged ≥60 years were living in the Makkah region during the time of data collection (22). Sample size calculation revealed that a sample of 271 was required to achieve sufficient statistical power based on a 90% confidence level, 5% margin of error, and 50% response distribution (23). The main inclusion criteria were an age ≥60 years and the ability to answer questions independently or with assistance from a caretaker. Participants were excluded if they had feeding tube, cognitive or physical disabilities, or were admitted to hospitals or institutions. This study was onducted in accordance with the guidelines of the Declaration of Helsinki, and all procedures were approved by the Biomedical Ethics Research Committee of King Abdulaziz University (Reference No. 503-21). Written informed consent was obtained from all participants. Data on all participants were collected *via* face-to-face interviews in public places, such as parks, malls, walking paths, Jeddah Corniche, and primary health care centers.

### 2.2. Data collection tools

#### 2.2.1. Demographic and anthropometric characteristics

Data on the demographic characteristics of the participants, such as age, sex, nationality, and marital status, were obtained. The following additional questions were asked: “Do you use food delivery applications?” (Answers: yes/no) and “Who prepares your food?” (Answers: myself/family/nobody). After the participants were interviewed, height (centimeters) and weight (kilograms) measurements were taken in light clothing using a standard scale. Body mass index (BMI), calculated by dividing the weight by height squared (kg/m^2^), was grouped according to the WHO categories (<18.5 kg/m^2^, underweight; 18.5–24.9 kg/m^2^, normal weight; 25.0–29.9 kg/m^2^, overweight; 30.0–34.9 kg/m^2^, obese; and ≥35 kg/m^2^, severely obese) (24). Given that only four and six participants were classified as being underweight and severely obese, respectively, we combined the data of participants who were underweight with that of those with a normal weight for the analysis. Similarly, the data of participants who were severely obese were combined with that of those who were obese.

A validated semi-structured questionnaire for data collection through face-to-face interviews was developed based on previous studies that used the Geriatric Depression Scale-Short Form (GDS-SF) (25, 26), Geriatric Oral Health Assessment Index (GOHAI) (27, 28), Mini Nutritional Assessment (MNA) scale (29, 30), Household Dietary Diversity (HDD) score (31, 32), and Eating Attitudes Test (EAT-26) (33, 34). To ensure reliability and validity, the researchers translated the tools into Arabic. These were subsequently sent to 15 participants for pre-testing, following which necessary changes were made to ensure that the questionnaire was culturally consistent.

#### 2.2.2. Geriatric depression scale-short form

This scale involved 15 questions that were highly associated with depressive symptoms based on the original GDS validation studies (25, 26). The GDS-15 examined the participants' mood. The given answers (yes/no) were based on how the participant felt in the past week. Depending on the question, 1 point was awarded to either a “yes” or “no” answer. Scores of 0 to 4 points were considered normal mental health, and 5 to 15 points were indicative of depression.

#### 2.2.3. Geriatric oral health assessment index

The GOHAI comprised 12 questions. Responses on a Likert scale were used to assess oral health problems that might affect daily life, including physical functions, such as eating, swallowing, and speaking; and psychosocial functions, such as worrying or concerns regarding oral health and self-image, in addition to self-consciousness of oral health and limitation of contact with people due to oral health problems. The scale also assessed pain and inconvenience. Responses were scored as follows: never = 5, seldom = 4, sometimes = 3, often = 2, and always = 1. Scores ranging from 57 to 60 were considered adequate oral health, scores ranging from 56 to 51 were considered moderate oral health, and scores ≤50 were considered poor oral health (27, 28).

#### 2.2.4. Mini nutritional assessment questionnaire

The MNA is a screening and assessment tool for older people that consists of 18 items. In this study, 16 items were used as the questions regarding mid-arm and calf circumferences were excluded. The tool is divided into two parts: the first part, which consists of questions regarding BMI, mobility, and weight loss, is used for screening, while the second part consists of questions regarding the living situation of participants, number of drugs used per day, self-perceptions of health and nutrition, and consumption of food and fluid. Each answer was scored and the sum of the points indicated the respondent's status. Participants with scores <17, 17–23.5, and ≥24 were considered malnourished, at risk of malnutrition, and well-nourished, respectively (29, 30).

#### 2.2.5. Eating attitudes test

Eating attitudes and behaviors were evaluated using the EAT-26 self-report questionnaire. The scale had three subscales with 26 questions about dieting, bulimia, and oral control. The questionnaire was related to beliefs, behaviors, and attitudes about food, weight, and body shape (33). Each item, except the behavior subscale, had six response options with scores ranging from 0 to 3 (always = 3, usually = 2, often = 1, sometimes = 0, rarely = 0, and never = 0). The behavior subscale had reversed scores (“never,” “once a month or less,” and “2–3 times a month” = 0; “once a week” = 1; “2–6 times a week” = 2; and “once a day or more” = 3). The overall score was equal to the sum of the scores of the 26 items. A score of ≥20 was characteristic of a disordered eating attitude (34).

#### 2.2.6. Household dietary diversity score

The HDD score was determined based on how many food groups were consumed in a week. The method was adapted from that of Clausen et al. (31). Twelve food groups were used to calculate the HDD score: (1) cereals; (2) roots and tubers; (3) vegetables; (4) fruits; (5) meat, poultry, and offal; (6) eggs; (7) fish and seafood; (8) legumes, nuts, and pulses; (9) milk and dairy products; (10) oil/fats; (11) sugar/honey; and (12) miscellaneous food types. For each food category, a score of 1 (if consumed) or 0 (if not consumed) was assigned. The HDD score, which ranged from 0 to 12, was determined by the total number of food categories consumed by the entire household. It was categorized based on the Food and Agricultural Organization of the United Nations' (32) recommendation: lowest dietary diversity (≤4 food groups), medium dietary diversity (>4 and <7 food groups), and high dietary diversity (≥7 food groups).

### 2.3. Statistical analyses

Descriptive statistics are presented as means and standard deviations. Linear regression models were used to assess the association of older age groups and nutritional status with oral health, depression, and eating disorder status, with age, sex, BMI, and marital status as covariates. Logistic regression models were constructed to determine the odds ratio (OR) between nutritional status and demographics (sex and age), BMI, oral health, depression, and eating disorders. In the logistic regression analysis, we combined the small number of participants with malnutrition (36 participants) with those at risk of malnutrition (146 participants) into a single group defined as the group at risk of malnutrition. Chi-square tests were used to analyze differences between nutritional status and comorbidities.

The life expectancy in Saudi Arabia in 2020 was estimated at 75 years (35); therefore, we opted for 70 years as the cutoff point to compare data between age groups, especially considering that most participants in this study were at the younger end of the age range of older people (<70 years). If we had opted for 75 years as the cutoff point, the comparison would not have been equivalent. Additionally, in Saudi Arabia, one in four adults have obesity or diabetes (36) (no data available for older people), and the prevalence of chronic diseases in Saudi men is high. One study reported that the percentage of participants aged ≥55 years with one and two or more chronic diseases is 31 and 34.5%, respectively. Furthermore, the prevalence of chronic diseases increases with age and obesity (37).

Statistical analyses were performed using SPSS version 28 (IBM Corp., Armonk, NY, USA). A *P*-value of 0.05 was set as the statistical significance level for all tests.

## 3. Results

Women represented 49% (men, 51%) of the participants enrolled in the study (Table 1). The mean age of the men and women was 69.2 ± 8.9 years and 68.4 ± 8.4 years, respectively (minimum 60 years and maximum 115 years for the total sample size). The mean BMI of the men and women was 28.4 ± 6.1 kg/m^2^ and 29.3 ± 6 kg/m^2^, respectively. According to the BMI categories, in the total sample, 25.1% of the participants had a normal weight, 36.2% were overweight, and 38.7% were obese; in men, the percentages were 26.8, 42.3, and 31.9%, respectively, and in women, 23.3, 30.8, and 45.9%, respectively. Of the participants, 73.8, 21.4, and 4.8% were married, widowed, and divorced, respectively. There were more married men (83.3%) than there were married women (63.9%; *P* < 0.001). The majority of the participants did not use food delivery services and their families prepared meals for them. This was particularly true for men where 92.8%, compared with 48.9% of women, reported that their families prepared meals for them (*P* < 0.001). Regarding health questionnaire responses, 72.3% of the participants had poor oral health, 43.2% felt depressed (ranging from mild to severe), and 67.2% were malnourished or at risk of malnutrition. Men tended to feel less depressed (*P* = 0.01) and were more at risk of malnutrition (*P* = 0.02) than were women. Among the participants at risk of malnutrition, 14.3% reported weight loss of more than 3 kg in the previous 3 months, 25.3% reported weight loss of 1–3 kg, 26.4% reported no weight loss, and 32.4% did not know if they had lost weight. However, only 20.3% had eating disorders. The mean HDD score of the study participants was 9.8 ± 1.7 and there were no significant differences between men (9.8 ± 1.8) and women (9.9 ± 1.6).

**Table 1:** Characteristics of the study participants.

**Characteristics**	**Total**	**Men (n=138)**	**Women (n=133)**	***P*** **-value**
Age (years)	68.8 ± 8.7	69.2 ± 8.9	68.4 ± 8.4	0.45
**Age range**				
60–69 years	161 (59.4)	78 (56.5)	83 (62.4)	0.27
70–79 years	75 (27.7)	44 (31.9)	31 (23.3)	
≥80 years	35 (12.9)	16 (11.6)	19 (14.3)	
**Body mass index (kg/m** ^2^ **)**				
BMI mean	28.8 ± 6.1	28.4 ± 6.1	29.3 ± 6	0.19
Normal weight	68 (25.1)	37 (26.8)	31 (23.3)	0.05
Overweight	98 (36.2)	57 (42.3)	41 (30.8)	
Obese	105 (38.7)	44 (31.9)	61 (45.9)	
**Nationality**				
Saudi	249 (91.9)	125 (90.6)	124 (93.2)	0.51
Non-Saudi	22 (8.1)	13 (9.4)	9 (6.8)	
**Marital status**				
Married	200 (73.8)	115 (83.3)	85 (63.9)	<0.001
Widowed	58 (21.4)	14 (10.1)	44 (33.1)	
Divorced	13 (4.8)	9 (6.5)	4 (3)	
**Use of food delivery**				
Yes	41 (15.1)	21 (15.2)	20 (15)	1.0
No	230 (84.9)	117 (84.8)	113 (85)	
**Food preparation**				
Myself	75 (27.7)	9 (6.5)	66 (49.6)	<0.001
Family	193 (71.2)	128 (92.8)	65 (48.9)	
Nobody	3 (1.1)	1 (0.7)	2 (1.5)	
**Oral health status**				
Poor	196 (72.3)	101 (77.7)	95 (76.6)	0.20
Moderate	55 (20.3)	29 (22.3)	26 (21)	
Adequate	3 (1.1)	0	3 (2.4)	
**Depression score**				
Normal	154 (56.8)	87 (63)	67 (50.4)	0.01
Mild	88 (32.5)	37 (26.8)	51 (38.3)	
Moderate	16 (5.9)	11 (8)	5 (3.8)	
Severe	13 (4.8)	3 (2.2)	10 (7.5)	
**EAT-26 score**				
Normal	209 (77.1)	109 (80.1)	100 (78.1)	0.76
Eating disorder	55 (20.3)	27 (19.9)	28 (21.9)	
**MNA score**				
Malnutrition	36 (13.3)	11 (8)	25 (18.8)	0.02
At risk of malnutrition	146 (53.9)	81 (58.7)	65 (48.9)	
Normal nutritional status	89 (32.8)	46 (33.3)	43 (32.3)	
Household dietary diversity score	9.8 ± 1.7	9.8 ± 1.8	9.9 ± 1.6	0.71

Data are presented as *n* (%), except for age and body mass index which are presented as mean ± standard deviation.

EAT-26, Eating Attitudes Test; MNA, Mini Nutritional Assessment.

*P*-values were calculated using an independent *t*-test for age, body mass index, and household dietary diversity score, and the Chi-square test for all categorical variables.

Table 2 compares the health status of older people aged 60–69 years, 70–79 years, and ≥80 years. Participants aged ≥80 years were at risk of depression (GDS score 7 ± 4.2) compared with those aged 70–79 years (GDS score 4.4 ± 2.9) and 60–69 years (GDS score 3.7 ± 3.1; *P* < 0.001) who had normal mean depression scores. Furthermore, the mean HDD score was significantly higher in participants aged 60–69 years (10.1 ± 1.7) than in those aged 70–79 years (9.4 ± 1.8) and ≥80 years (9.7 ± 1.4; *P* = 0.04).

**Table 2:** Association of age with nutritional status and other health conditions.

**Variables**	**60–69 years**	**70–79 years**	**≥80 years**	***P*** -value^*^
Oral health score	42.7 ± 9.1	42.3 ± 8.5	40.5 ± 9.1	0.65
Depression score	3.7 ± 3.1	4.4 ± 2.9	7 ± 4.2	<0.001
Eating disorder score	14.5 ± 12.6	12.6 ± 9.3	11.6 ± 8.8	0.11
Nutritional status score	21.9 ± 4.1	21.6 ± 4.6	19.2 ± 4.4	0.05
Household dietary diversity score	10.1 ± 1.7	9.4 ± 1.8	9.7 ± 1.4	0.04

* *P*-values were calculated using linear regression adjusted for sex, body mass index, and marital status.

Data are presented as mean ± standard deviation.

The association between nutritional status and other health conditions is shown in Table 3. Nutritional status was significantly associated with oral health (*P* < 0.001), depression (*P* < 0.001), and eating disorder scores (*P* = 0.002). Participants with malnutrition or at risk of malnutrition were depressed, had a poor oral health status, and had a higher risk of developing eating disorders compared with participants with a normal nutritional status.

**Table 3:** Association between nutritional status of older people and other health conditions.

**Variables**	**Malnourished (*n* = 36)**	**At risk of malnutrition (*n* = 146)**	**Normal nutritional status (*n* = 89)**	***P*** -value^*^
Oral health score	38.3 ± 8.8	40.5 ± 8.7	46.6 ± 7.5	<0.001
Depression score	8.1 ± 3.5	4.4 ± 2.9	2.6 ± 2.4	<0.001
Eating disorder score	16.5 ± 10.8	15.1 ± 12.9	10.1 ± 7.5	0.002
Household dietary diversity score	9.6 ± 1.5	9.7 ± 1.8	10.1 ± 1.6	0.11

* *P*-values were calculated using linear regression adjusted for sex, age, body mass index, and marital status.

Data are presented as mean ± standard deviation.

Table 4 shows the adjusted ORs of nutritional status derived from the logistic analysis. The factors associated with higher odds of malnutrition were overweight or obesity (OR, 2.44; 95% confidence interval [CI], 1.25–4.77; *P* = 0.009), oral health (OR, 4.43; 95% CI, 2.28–8.58; *P* < 0.001), depression (OR, 0.21; 95% CI, 0.11–0.42; *P* < 0.001), and eating disorders (OR, 0.27; 95% CI, 0.11–0.62; *P* < 0.002). Additionally, older individuals with peripheral vascular disease (*P* = 0.009), asthma (*P* = 0.04), and hypertension (*P* < 0.001) had a significant risk of malnutrition. Among older participants, overweight or obesity, oral health, and depression were factors associated with an increased risk of being malnourished or at risk of malnutrition when compared with participants with a normal nutritional status (all *P* ≤ 0.02; data not presented).

**Table 4:** Odds ratios for the risk of malnutrition among the participants.

**Variables**	**Adjusted OR (95% CI)**	***P*** -value^*^
**Body mass index**		
Normal	Reference	
Overweight or obese	2.44 (1.25–4.77)	0.009
**Oral health**		
Poor	Reference	
Adequate	4.43 (2.28–8.58)	<0.001
**Depression**		
Normal	Reference	
Depressed	0.21 (0.11–0.42)	<0.001
**Eating disorders**		
Normal	Reference	
Has an eating disorder	0.27 (0.11–0.62)	0.002
**Comorbidities**		
Healthy	Reference	
Peripheral vascular disease	0.13 (0.03–0.60)	0.009
Healthy	Reference	
Asthma	0.41 (0.17–0.97)	0.04
Healthy	Reference	
Diabetes	0.74 (0.43–1.28)	0.28
Healthy	Reference	
Hypertension	0.26 (0.14–0.47)	<0.001

* *P*-values were calculated using logistic regression adjusted for age, body mass index, and marital status, depending on the variable analyzed.

OR, odds ratio; CI, confidence interval.

The frequency of comorbidities among older people based on nutritional status is shown in Table 5. The frequency of congestive heart failure (30.6%), peripheral vascular disease (30.6%), cerebrovascular disease (11.1%), asthma or chronic lung disease (36.1%), liver disease (17.1%), peptic ulcer disease (47.2%), Alzheimer's disease or dementia (30.6%), rheumatic disease (33.3%), hypertension (74.3%), peripheral skin ulcer (25%), and depression (41.2%) was significantly higher among participants with malnutrition than among those at risk of malnutrition or with an adequate nutritional status.

**Table 5:** Frequency of comorbidities stratified by nutritional status.

**Comorbidities**	**Malnourished (*n* = 36)**	**At risk of malnutrition (*n* = 146)**	**Normal nutritional status (*n* = 89)**	***P*** -value^*^
Myocardial infarction	7 (19.4)	21 (14.5)	7 (7.9)	0.15
Congestive heart failure	11 (30.6)	25 (17.2)	9 (10.1)	0.02
Peripheral vascular disease	11 (30.6)	16 (11)	2 (2.2)	<0.001
Cerebrovascular disease	4 (11.1)	4 (2.8)	1 (1.1)	0.01
Hemiplegia or paraplegia	1 (2.8)	2 (1.4)	0	0.36
Asthma or chronic lung disease	13 (36.1)	23 (15.9)	8 (9)	<0.001
Diabetes without chronic complications	19 (52.8)	62 (42.8)	36 (40.4)	0.44
Renal disease, severe	1 (2.8)	5 (3.5)	3 (3.4)	0.86
Liver disease, moderate to severe	6 (17.1)	9 (6.2)	1 (1.1)	0.02
Peptic ulcer disease	17 (47.2)	23 (16)	10 (11.2)	<0.001
Alzheimer's disease or dementia	11 (30.6)	6 (4.1)	2 (2.2)	<0.001
Rheumatic disease	12 (33.3)	20 (14.1)	9 (10.1)	0.004
HIV or AIDS	2 (5.6)	2 (1.4)	0	0.06
Hypertension	26 (74.3)	74 (51.7)	25 (28.1)	<0.001
Peripheral skin ulcer	9 (25)	9 (6.3)	2 (2.2)	<0.001
Depression	14 (41.2)	18 (12.4)	3 (3.4)	<0.001
Cancer	2 (5.7)	6 (4.2)	0	0.11

* *P*-values were calculated using the Chi-square test.

Data are presented as *n* (%).

HIV, human immunodeficiency virus; AIDS, acquired immunodeficiency syndrome.

## 4. Discussion

This cross-sectional study aimed to investigate the factors associated with the nutritional status of older people in the Makkah region of Saudi Arabia. The study revealed that malnutrition among older people in Saudi Arabia was associated with oral health, HDD score, overweight or obesity, and depression. Furthermore, we found that compared with participants <80 years of age, participants ≥80 years were at higher risk of depression. In addition, a high percentage of the participants was obese, with a higher proportion of women being obese, and a higher proportion of men being overweight. Overall, this study revealed that older people at risk of malnutrition had significantly higher rates of peripheral vascular disease, asthma or chronic lung disease, peptic ulcers, Alzheimer's disease or dementia, hypertension, and depression than did participants with an adequate nutritional status. Thus, the study results support our hypothesis that older people with malnutrition are at higher risk of different diseases.

In this study, the proportion of participants with or at risk of malnutrition was 67%, which is consistent with the findings of studies from Australia (4) and India (38) that reported a proportion of 50–55%. In contrast, in Spain (6), Turkey (39), Sweden (40), and Hong Kong (12), the percentages were approximately one-third of those found in our study (27, 18.6, 17, and 1.1%, respectively), which may be explained by the high standard of health care for older people in these countries. The reported malnourished status could have resulted from unhealthy dietary choices and inadequate consumption of nutrient-rich foods. Another reason could be that systemic inflammation caused by obesity affects the absorption, distribution, and excretion of nutrients, thereby altering micronutrient metabolism (41). A study conducted among 782 French older adults (aged ≥65 years) found that 18% of overweight and 29% of obese individuals were at risk of undernutrition (42). Furthermore, in contrast with studies from India (6), Bangladesh (19), and France (42) that reported higher malnutrition rates among women than among men, we found that more men than women were at risk of malnutrition. A possible explanation for this might be that only 49% of women but the majority of men (92%) in our study were dependent on their families for food preparation. Previous studies have shown that poor health, a higher risk of diseases, and insufficient nutrient intake in older people may increase the risk of malnutrition. In addition, there are several factors associated with malnutrition or the risk of malnutrition, such as socioeconomic factors (low income, living alone [being single, widowed, or divorced], low education level), eating difficulties and disorders, and depression, especially in older people (40, 43–46). In the present study, the percentage of married men (83.3%) was higher than that of married women (63.9%); however, 21.4% and 4.8% of the participants were widowed and divorced, respectively. According to Bakker et al. (43), such participants may live alone without a helper, which may increase their risk of malnutrition compared with those who live with their families. Several studies have found that a significant percentage of older people at risk of malnutrition have a low socioeconomic status, such as low income and no education (13, 19), which influences dietary intake and eating patterns (13); however, we did not assess these factors in our study. These factors are related to age-related changes in taste, smell, dental health, and biological and physical activities, which can reduce the quality and quantity of nutrient intake and increase the risk of malnutrition in older people (6, 47, 48).

Eating difficulties are an important risk factor for malnutrition and can be caused by gradual muscle degradation, loss of teeth, and loss of motor coordination, leading to difficulties with the handling of food, putting food in the mouth, and chewing and swallowing (dysphagia) (39, 44). These difficulties can lead older people to opt for foods that are low in fiber and nutrients (44), which may increase their risk of malnutrition. In this study, 72.3% of the participants reported that they had poor oral health and 67% were at risk of malnutrition. In older people, There was a significant association between older people with poor oral health (chewing or swallowing difficulties) and being malnourished or at risk of malnutrition, which is consistent with the results of a Lebanese study (45) that used the GOHAI score and reported that 75% of older people had poor oral health and 41.5% were malnourished or at risk of malnutrition. Similarly, studies in Bangladesh (46) and India (38) have reported that 63% and 59.5% of older people who were malnourished, respectively, had poor oral health, whereas in Japan (49), the result (29.6%) was less than that reported in our study.

The mean HDD score was significantly higher in participants aged 60–69 years compared to in those aged ≥70 years; it was also high in comparison with that found in developing countries where there is limited HDD with food options mainly based on starchy staple foods (50). For example, the HDD score among the older population was found to be low in Sri Lanka (51), South Africa (52), and China (53). In contrast, the HDD score among older women was high in the United States (54). The high HDD score found in this study might be a result of high food availability and variability for older adults. In Saudi Arabia, family support and social relationships may play an important role in increasing HDD—family, friends, and neighbors can provide social and economic support (55). In addition, families in Saudi Arabia function as a unit socially and emotionally. Younger members of the family serve older members, and the concept of sending an older member to a nursing home is unacceptable in Saudi culture (56).

After 50 years of age, various changes occur in body composition, such as increases in fat mass and decreases in lean mass (sarcopenia) (57). Therefore, the basal metabolic rate decreases by ~15% between the ages of 30 and 80 years, which consequently reduces energy requirements. However, appetite and nutrient requirements remain the same or increase, which can further lead to weight gain during this age period (58). The frequency of obesity, which is associated with several chronic diseases, such as diabetes, hypertension, and cardiovascular disease, increases in older people aged between 60 and 70 years, particularly in women (59, 60). A high percentage of our study participants were obese (38.7%) and overweight (36.2%), and obesity was more prevalent in women (45.9%) than in men (31.9%), while more men were overweight (41.3%) than were women (30.8%). This is in contrast with the results of other studies showing that older women often have eating disorders because they are still concerned about their body shape (61, 62). In relation to other countries, similar trends in BMI status and the prevalence of overweight have been observed in older men (49 and 31.5%, respectively) and older women (39.8 and 40.8%, respectively) in Spain (63), while a comparatively lower prevalence of obesity has been observed in older people in India (32.5% in both men and women) (6). Our finding was also consistent with that of a study in Scotland (64), which found that obesity prevalence was particularly higher in older women. In the present study, participants at risk of malnutrition had low HDD scores compared with those with a normal nutritional status. Lv et al. found an inverse relationship between HDD score and mortality risk in older adults, with a 44% lower mortality risk among participants with the highest HDD score (65). A study in the United States found a correlation between HDD and BMI in women (54). Furthermore, in Zambia, most older adults were found to have a low HDD and a poor nutritional status (66).

The risk of malnutrition may increase in older individuals who have at least one chronic disease, such as respiratory disease, arthritis, stroke, depression, dementia, gastric diseases, or cancer (59, 67). Moreover, the use of medications for treating these conditions may affect the appetite and swallowing function of older people and reduce the quantity and quality of food consumption and nutrient bioavailability, thereby increasing the risk of malnutrition (59, 67). Our study reported that older adults with peripheral vascular disease, asthma, and hypertension had a significant risk of malnutrition. We also found that 55% of older Saudis were malnourished and diagnosed with hypertension, whereas studies in Algeria (60) and Bangladesh (19) reported that 39.8 and 88.9% of older people, respectively, were malnourished and diagnosed with hypertension. On the other hand, our study showed that 19.8% of the participants who were at risk of malnutrition had been diagnosed with asthma or chronic lung disease, significantly less than that reported in Bangladesh (79%) (19).

A total of 15% of the participants at risk of malnutrition had peripheral vascular disease, which is relatively high. However, studies have indicated that diabetes and heart failure are important risk factors for peripheral vascular disease (58), and almost a half and a quarter of the older people in our study were diagnosed with diabetes and heart failure, respectively. This may explain the percentage of peripheral vascular disease in our cohort (30.6%). In a study by Saquib et al. (37), conducted in the Al-Qassim region of Saudi Arabia, the prevalence of chronic diseases including hypertension, diabetes, heart disease, asthma, ulcers, and cancer in Saudi men was 71.3, 27.3, 16.4, 9.7, 8.9, and 2.0%, respectively. The participants in our study were relatively young (68.8 ± 8.7 years) based on the WHO definition of the young-old (60–74 years) (58); this group is usually healthy and independent. Furthermore, this study excluded participants with feeding tube and physical disabilities, as well as those who were admitted to hospitals or in-care institutions. Nevertheless, the prevalence of several diseases, such as asthma or chronic lung disease, Alzheimer's disease or dementia, hypertension, and depression, was relatively high in our study compared with studies conducted in other countries.

Regarding mental health status, the frequency of Alzheimer's disease or dementia and depression was relatively low in our study (9.3 and 17.6%, respectively). A Swedish systematic review reported that 1.5% and 35% of older people aged 64–69 and 90–94 years, respectively, have dementia and are at risk of malnutrition (68). Furthermore, our results showed an association between depression and the risk of malnutrition among older people; this association was more significant in those aged ≥80 years (mild depression) than in those aged <80 years (normal depression status). Similarly, Iranian (69) and Bangladeshi (19) studies have confirmed that malnutrition is significantly higher among older people with depression, with further studies in Bangladesh and Japan (19, 47) reporting the odds of malnutrition among older people who are depressed to be 15.6 and 6.3 times higher, respectively, than those who are not depressed (compared with 10 times higher in our study). Furthermore, among the participants at risk of malnutrition in our study, men tended to be less depressed than were women, which is in agreement with 69 among the 85 studies included in a review by Girgus et al. (70). Mantzorou et al. (71) also concluded that malnutrition is more common in older people who have cognitive decline and depressive symptoms. These results may be explained by the effect of depression on appetite and eating behaviors (69).

A health care system aimed at the older population has been developed in various countries. In European countries, the WHO has been working with governments to establish five priority intervention strategies: the prevention of falls and infectious diseases, promotion of physical activity, support for home care, and capacity building among health and social care workers (72). In Saudi Arabia, the Ministry of Health has established “The Home Care Program for Older Adults” to oversee all home medical services (73). Consequently, all stakeholders were required to contribute to developing policies, programs, and interventions for healthy older adults to maintain independence and remain as active as possible (74). In general, these health care systems can help to improve the health of older adults and reduce the occurrence of malnutrition in Saudi Arabia.

This study has several limitations. Despite the importance of socioeconomic level in terms of nutritional status, factors including economic status, educational level, and urban-rural were not assessed in this survey. Data on food intake, clinical signs, and laboratory investigations were also not collected to determine other factors of malnutrition. Furthermore, participants were from the Makkah region of Saudi Arabia, which limits the generalizability of the findings to the whole Kingdom. Further studies with larger sample sizes that recruit participants from all regions of Saudi Arabia are required to verify our findings. Moreover, the current study used convenience sampling which is a non-probability sampling method that might introduce selection bias, as those who were engaged in the research matter might have been more interested in participating. Nevertheless, this study is one of the few focused on the factors associated with nutritional status in Saudi Arabia, which is its the main strength. Another strength of this study is the use of several validated questionnaires, and the fact that anthropometric indices (weight and height) were measured and not self-reported. Additionally, there is a lack of research focusing on the nutritional and health statuses of older people globally, and particularly in Saudi Arabia, which highlights the importance of this study. Our findings can help to develop strategies to improve nutritional status and prevent malnutrition among older people, thereby reducing treatment costs.

## 5. Conclusion

Our study is one of the few studies assessing the factors associated with the nutritional status of older people, especially in Saudi Arabia. It can be concluded that there is a high proportion of malnutrition in the older population of the Makkah region of Saudi Arabia. The majority of older people of both sexes were overweight or obese, with a high risk of malnutrition. Oral health, depression, and eating disorders were factors related to the nutritional status of older people in Saudi Arabia. Among individuals with malnutrition, there was a significantly higher prevalence of peripheral vascular disease, asthma or chronic lung disease, peptic ulcers, Alzheimer's disease or dementia, hypertension, and depression than among individuals at risk of malnutrition and those with a healthy nutritional status. Generally, older people and their caretakers did not recognize symptoms of malnutrition in the early stages. Therefore, they did not seek treatments such as modified food or supplements until several conditions and complications had developed. The risk of malnutrition among older people in Saudi Arabia and globally can be reduced by regular nutritional screening and subsequent designing of nutritional interventions for timely treatment to prevent complications. The factors related to malnutrition among older people in Saudi Arabia should be studied in the future due to the increased proportion of this age group in this population group.

## Data availability statement

The raw data supporting the conclusions of this article will be made available by the authors, without undue reservation.

## Ethics statement

The studies involving human participants were reviewed and approved by Biomedical Ethics Research Committee of King Abdulaziz University (Reference No. 503-21). The patients/participants provided their written informed consent to participate in this study.

## Author contributions

Conceptualization: MA. Methodology: MA and IS. Formal analysis: IS. Data collection, writing—original draft preparation, reviewing, and editing of the manuscript: MA, IS, NAlm, and NAlj. All authors have read and agreed to the published version of the manuscript.
